# A study of voice and non-voice processing in Prader-Willi syndrome

**DOI:** 10.1186/s13023-020-1298-8

**Published:** 2020-01-20

**Authors:** Kuzma Strenilkov, Jimmy Debladis, Juliette Salles, Marion Valette, Carine Mantoulan, Denise Thuilleaux, Virginie Laurier, Catherine Molinas, Pascal Barone, Maïthé Tauber

**Affiliations:** 1 Brain & Cognition Research Center (CerCo), University of Toulouse Paul Sabatier, Toulouse, France; 2 Brain & Cognition Research Center (CerCo), CNRS, Toulouse, France; 3 ENT Department, Purpan Hospital, Toulouse, France; 4 Prader-Willi Syndrome Reference Center, Children’s Hospital-INSERM-University of Toulouse Paul Sabatier, Toulouse, France; 5 Marine Hospital, Hendaye, France; 6 Toulouse Purpan Physiopathology Center, INSERM-University of Toulouse Paul Sabatier, Toulouse, France

**Keywords:** Prader-Willi syndrome, Voice processing, Social interactions, Autism spectrum disorder

## Abstract

**Background:**

Prader-Willi syndrome (PWS) is a rare and complex neurodevelopmental disorder of genetic origin. It manifests itself in endocrine and cognitive problems, including highly pronounced hyperphagia and severe obesity. In many cases, impaired acquisition of social and communication skills leads to autism spectrum features, and individuals with this syndrome are occasionally diagnosed with autism spectrum disorder (ASD) using specific scales. Given that communicational skills are largely based on vocal communication, it is important to study human voice processing in PWS.

We were able to examine a large number of participants with PWS (*N* = 61) recruited from France’s national reference center for PWS and other hospitals. We tested their voice and nonvoice recognition abilities, as well as their ability to distinguish between voices and nonvoices in a free choice task. We applied the hierarchical drift diffusion model (HDDM) with Bayesian estimation to compare decision-making in participants with PWS and controls.

**Results:**

We found that PWS participants were impaired on both voice and nonvoice processing, but displayed a compensatory ability to perceive voices. Participants with uniparental disomy had poorer voice and nonvoice perception than participants with a deletion on chromosome 15. The HDDM allowed us to demonstrate that participants with PWS need to accumulate more information in order to make a decision, are slower at decision-making, and are predisposed to voice perception, albeit to a lesser extent than controls.

**Conclusions:**

The categorization of voices and nonvoices is generally preserved in participants with PWS, though this may not be the case for the lowest IQ.

## Background

Prader-Willi syndrome (PWS) is a rare genetic disease that was first described in 1956. It is caused by the absence or inactivation of paternal genes in the 15q11.2-q13 region of chromosome 15. The absence of gene expression is due to one of the following genetic subtypes: q11–13 de novo deletion on chromosome 15 of paternal origin (DEL; 60% incidence); chromosome 15 maternal uniparental disomy (UPD; 35%) [1]. Nowadays, diagnosis is made during the first months of life, and the prevalence of each genetic subtype currently stands at 50% for DEL and 50% for non-DEL. The main diagnostic criteria for PWS are severe hypotonia at birth, associated with difficulty sucking and swallowing, which causes low weight gain with failure to thrive [2]. Around the age of 2–3 years, although no change in food intake is observed [3], excessive weight gain occurs, followed by a sudden behavioral change that manifests itself as eating disorders leading to the hyperphagia that characterizes this disease. During early childhood and adolescence, cognitive disorders and a mild or moderate mental deficit emerge alongside this behavioral disturbance. Although an overall delay in the acquisition of certain skills (motor, communication, cognitive) often leads to the behavioral alterations similar to autism spectrum disorder (ASD), it is only fully diagnosed in 20–40% of PWS cases [4, 5].

Given the similarities with ASD in terms of social behavioral alterations, it is important to study participants’ communication skills, which include both human voice and face processing [6]. Voices, just like faces, can tell us a great deal about individuals. Beyond the linguistic aspect, voices make it possible to identify the type of person, as well as that person’s age, identity, and sometimes corpulence [7]. Prosody also gives us access to individuals’ emotions and states of mind. Although there is a large body of knowledge about face processing disturbances in ASD, some studies have also demonstrated that voice processing can be impaired in autism [8, 9]. This voice processing difficulty could cause social interaction disorders or be linked to the lack of social motivation found in autism [10].

Little is known about voice processing in PWS. These participants are described as having difficulty discriminating vocal sounds [11], but it is still unclear whether voices, being socially important entities, are considered as a separate category, as is the case in a healthy population. We therefore set out to explore the voice recognition skills of participants with PWS by administering a simple two-alternative forced-choice task (2FAC) adapted to their intellectual disability (ID). Importantly, we were able to collect the data of more than 60 participants with this rare pathology. This large cohort of participants has also allowed us to analyze and compare the genetic subtypes that are differently impaired on face processing [12].

One of the aims of our study was to evaluate the origins of social information processing deficits in PWS. The large amount of data we collected allowed us to apply a specific model (hierarchical drift diffusion model, HDDM [13]; to clearly differentiate any sensorimotor deficit from a cognitive deficit related to decision-making in a 2FAC protocol. In most psychological tests of sensory processing to date [11], participants with PWS have systematically had longer reaction times (RTs), which have been attributed to early developmental deficits in sensorimotor integration skills [14]. The HDDM would allow us to study the neurocognitive implementation of psychological decision making processes. It might help us to decipher whether the slower RTs of participants with PWS can be attributed to particular features of their cognitive processing, such as the need to accumulate information in order to make choices.

In addition, there is now a large body of evidence that when social cognition is evaluated in ASD, participants can present performance levels close to those of typically developed (TD) controls, reflecting the adoption of adaptive strategies [15]. In some cases, these results can be explained by the fact that the experimental protocol elicited the explicit use of social cognition mechanisms. When it comes to dissociating implicit from explicit mechanisms, a free sorting task (FST) constitutes a good alternative to 2FAC protocols, as it can even be performed by young children [16]. We developed an FST with different types of natural environmental sounds, including voice sounds. In this test, participants can group items on the basis of either perceptual criteria (pitch, intensity, rhythmicity, etc.) or semantic criteria (everyday listening). In the latter case, categorization relies on the internalization of auditory objects, but this can be impaired in participants with disorders such as ASD [17]. In addition, the FST protocol makes it possible to analyze participants’ hierarchical representation of natural sounds, and yields a clear assessment of their implicit categorization.

## Results

### Hit rates and reaction times

**Fig. 1:**
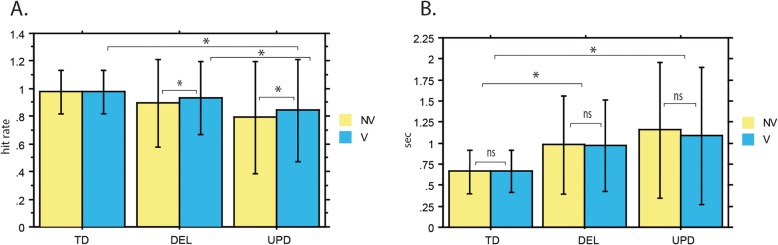
Performance on voice (V) and nonvoice (NV) processing. This figure illustrates the performance of typically developed (TD) subjects, participants with the chromosome 15 deletion (DEL) and uniparental disomy (UPD) in terms of their hit rates (**a**) and reaction times (**b**) for Voice (V) or Non-voice (NV) stimuli. Concerning hit rates, participants with PWS, especially UPD participants, were deficient in the recognition of voices and non-voices. This deficit was slightly weaker for voices than for non-voices in both genetic subgroups. To avoid clutter, only significant effects for voices are indicated in the figure as (*). The deficit in hit rates was accompanied by significantly longer reaction times with no difference between voices and non-voices

Voice identification is a relatively simple and easy task, and controls achieved a high level of performance (hit rate of over 97% for both vocal and nonvocal stimuli). An inspection of performance data indicated that PWS participants exhibited deficits in this task. In both PWS participant subgroups, performances were below 95% on average, but we observed considerable variability in individual performance levels. To pinpoint the differences in performance between the PWS participant subgroups and the control group, we entered their hit rates into the general linear mixed-effect model. This allowed us to estimate performances for both voices and nonvoices within each group, and differences in performance between the groups for each type of stimulus (Fig. 1).

The analysis of hit rates (Fig. 1a) revealed a significant effect of group (*p* < 0.001). Using post hoc tests to explore the effect of group, we found that for voices, UPD participants had a lower mean hit rate than controls (84% vs. 97%, *p* < 0.001). However, the difference with controls for voice perception only tended toward significance for DEL participants (92% vs. 97%, *p* = 0.0522). Thus, compared with controls, UPD participants had a pronounced deficit for voice perception, whereas this deficit was quite weak for the DEL participants. Importantly, we also observed a significant difference on voices when we directly compared the PWS participant subgroups: UPD had lower hit rates for voices than DEL (84% vs. 92%, *p* < 0.001). This confirmed that UPD participants have a greater voice perception deficit than DEL participants.

Concerning nonvoices, the mean hit rate was significantly lower for DEL (89%, *p* < 0.001) and UPD (79%, *p* < 0.001) participants than for controls (97%) (Fig. 1a). This means that both PWS subgroups were deficient in nonvoice perception. Similarly to the above results for voices, UPD participants also had a lower hit rate than DEL participants for nonvoices (79% vs. 89%, *p* < 0.001). The UPD participants therefore had a more pronounced deficit than the DEL participants for both types of stimulus.

In addition to the significant main effect of group, the analysis of hit rates revealed a significant effect of stimulus (*p* < 0.001), as well as a significant Group x Stimulus interaction (*p* < 0.05) (Fig. 1a). To see the directions of these effects, we examined the interaction further by running post hoc comparisons. These indicated that the difference in hit rates between voices and nonvoices was not significant for controls (*p* = 0.842), whereas hit rates were significantly higher for voices than for nonvoices in both the DEL (*p* < 0.001) and UPD (*p* < 0.01) participant subgroups. Thus, the equality of performance for voices and nonvoices in controls was not observed in PWS participants. Both PWS subgroups performed more poorly on nonvoices than on voices, possibly reflecting more pronounced compensation effects for such socially important stimuli as voices.

We also searched for correlations between hit rates and general intelligence (IQ) or clinical (DBC) scores, but none were found.

As previously observed adopting a simple discrimination approach [11], participants with PWS responded much more slowly to the vocal/nonvocal stimuli. Overall, mean RTs were about 50% longer for PWS participants than for controls (1005 ms vs 660 ms) but the only significant effect was for group (*p* < 0.001) (Fig. 1b). For both voices and nonvoices, post hoc tests showed that DEL and UPD participant subgroups had longer RTs than controls (*p* < 0.001). PWS participants’ longer RTs, taken together with their lower hit rates, may reflect a deficit in the perception of voices and nonvoices. Furthermore, for both voices and nonvoices, UPD participants had longer RTs than DEL participants did (*p* < 0.05). UPD participants also had lower hit rates than DEL participants, so the longer RTs support the notion of a more pronounced deficit for voice and nonvoice perception in UPD participants.

### HDDM parameters

**Fig. 2:**
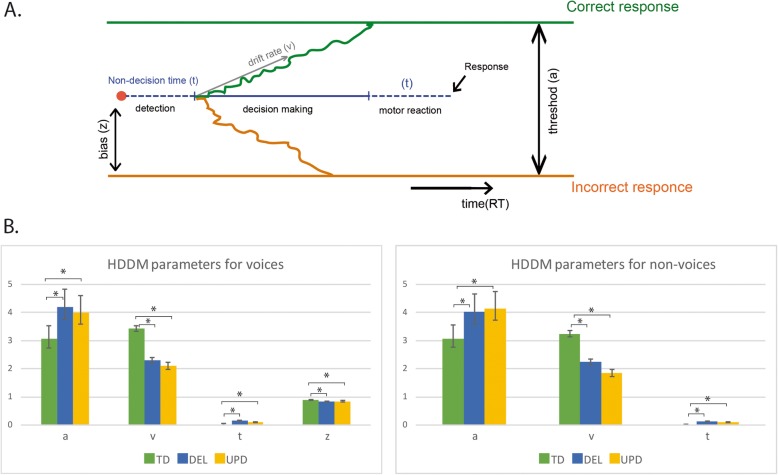
Hierarchical drift diffusion model for voices and nonvoices. This figure provides a scheme of the Bayesian estimation of the drift-diffusion model (**a**). The drift-diffusion model makes it possible to assess how much information individuals need to make a decision, thus separating decision criteria from non-decision processes. Different parameter of decision making are obtained (see Methods): the threshold (a); the drift rate (v) the non-decision reaction time (t) and the initial bias (z). The differences between the groups of participants concerning these parameters of the model are provided and compared in (**b**). For both voice and non-voice identification, DEL and UPD participants exhibited a similar pattern of changes in model parameters with respect to the TD participants. They had a higher threshold, a lower drift rate and longer non-decision times than controls. Their bias for voices was lower than in controls. Other conventions as in Fig. 1

The Bayesian estimation of the HDDM (Fig. 2a) indicated that in order to make a decision, participants had to integrate a certain amount of information represented by a threshold, at a specific speed represented by a *drift rate*. *Nondecision time* corresponded to the time required to execute the motor control and detect the stimulus (i.e., excluding time involved in decision-making). The total RT can be regarded as a combination of these parameters. We estimated the differences in the HDDM parameters between the groups of PWS participants and controls. For voice identification, participants with PWS had a higher threshold, lower drift rate, and longer nondecision time than controls (Fig. 2b). The threshold of participants with PWS was about 30% higher than that of controls, indicating that they needed to accumulate more information before making a decision about a perceived stimulus. This accumulation also took longer, as expressed by the lower drift rate. However, nondecision time was also longer, indicating slower stimulus perception and response execution in PWS participants. When all these parameters are considered together, it is clear that the greater deficit observed in the voice discrimination task originated from differences with controls on both threshold and drift rate values.

A similar pattern of HDDM parameters was found for nonvoices, as PWS participants also had a higher threshold, lower drift rate, and longer nondecision time than controls (Fig. 2). Thus, participants with PWS used similar strategies for both voice and nonvoice perception.

Regarding the distinction between participants with DEL or UPD, no significant differences were found on the HDDM parameters.

In a separate model including voices and nonvoices, we found an initial bias toward voices in all the groups, but this bias was higher in controls than in participants with PWS (Fig. 2b, *z* values). This means that controls were automatically more predisposed to perceive voices - a bias that was not so strongly present in participants with PWS.

### Sound categorization task

**Fig. 3:**
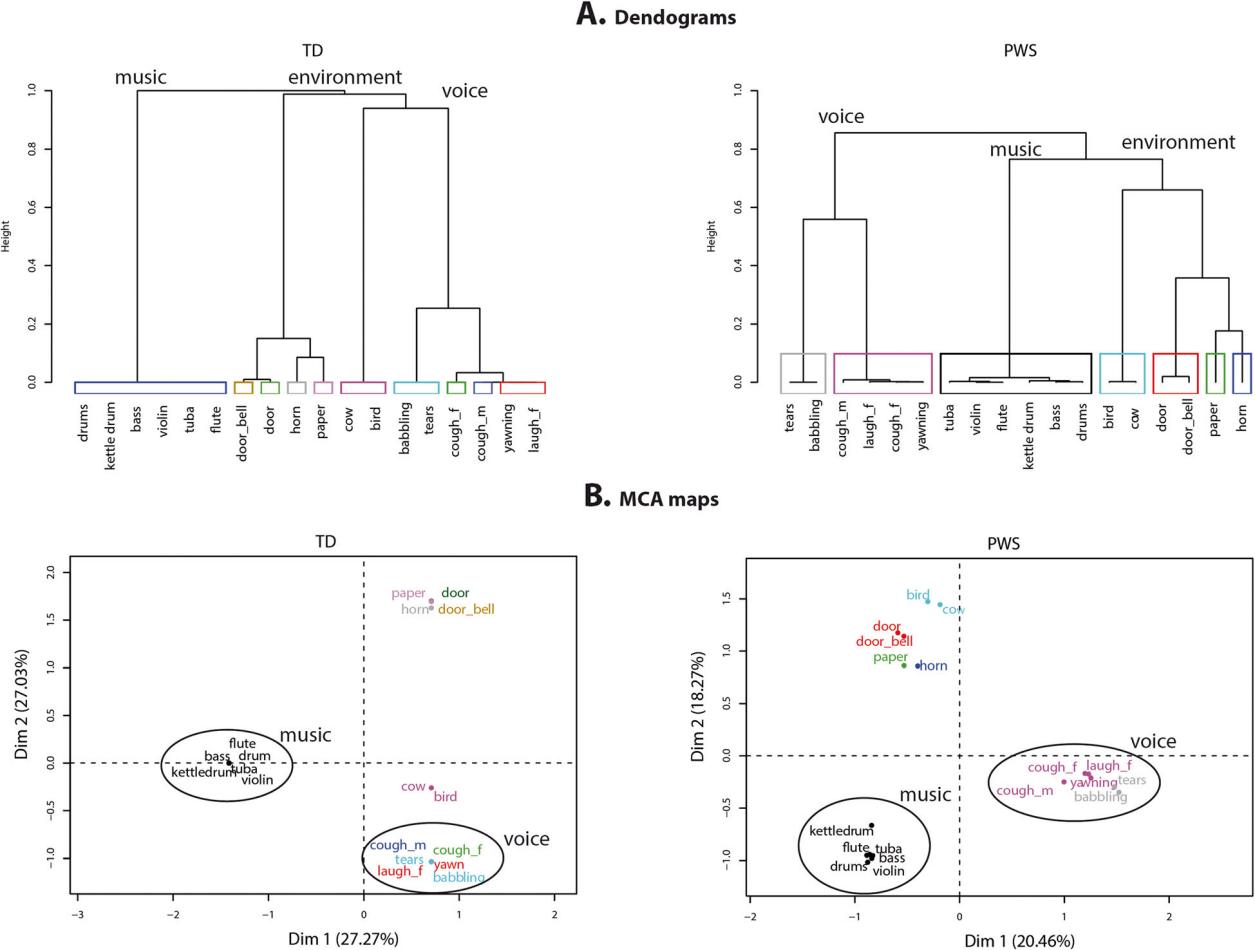
Dendograms and MCA maps for sound categorization by participants with Prader-Willi syndrome and typically developed controls. In **a**, the branches corresponding to the largest categories are named. In **b**, the circled sound categories are voice and musical instruments. Both the tree diagrams (**a**) and MCA maps (**b**) showed that participants with PWS created the similar voice, instruments and environmental categories

In line with our previous results, controls’ categorization was predominantly based on semantic information, as a result of identifying a sound source. The tree diagrams (Fig. 3a) show that controls categorized sounds as vocal, musical, or environmental sounds. Interestingly, the diagram showing categorization by participants with PWS is very similar, as the same three categories emerge from the first branches. This suggests that PWS participants made their categorization based on the same semantic criteria and according to the same hierarchical order. However, in the diagram, the between-category distance is clearly greater for controls than for participants with PWS, indicating that controls adopted a more homogeneous categorization strategy, where the categories were more clearly separated.

M ultiple correspondence analysis (MCA) was applied to the categorization performed by the two groups of participants in order to assess their overall categorization strategies. Analysis was restricted to the dimensions that explained the most variance within the original data, and we only report results for the first two dimensions, which together accounted for 54% of the total variance for controls and 39% for participants with PWS. In the MCA maps (Fig. 3b), the first dimension clearly reflects a distinction between voices and instruments for both the PWS participant and control groups. However, whereas the second dimension reflects a division between environmental sounds and animal sounds for controls, participants with PWS grouped animal and environmental sounds together. This absence of segregation of animal vocalizations by PWS participants appears to be the main difference in strategy between the two groups.

Furthermore, in order to analyze how clearly human voice stimuli were categorized, we compared the Euclidean distances between the categories of voices and other sounds (nonvoices). These comparisons revealed no significant difference on distances between controls and PWS participants (*p* > 0.05). However, within the voice category, as well as within the other categories formed by the participants with PWS, the distances between the stimuli were significantly greater than they were within the categories formed by controls (*p* < 0.05). This means that PWS participants grouped together sounds with greater variability than controls.

**Fig. 4:**
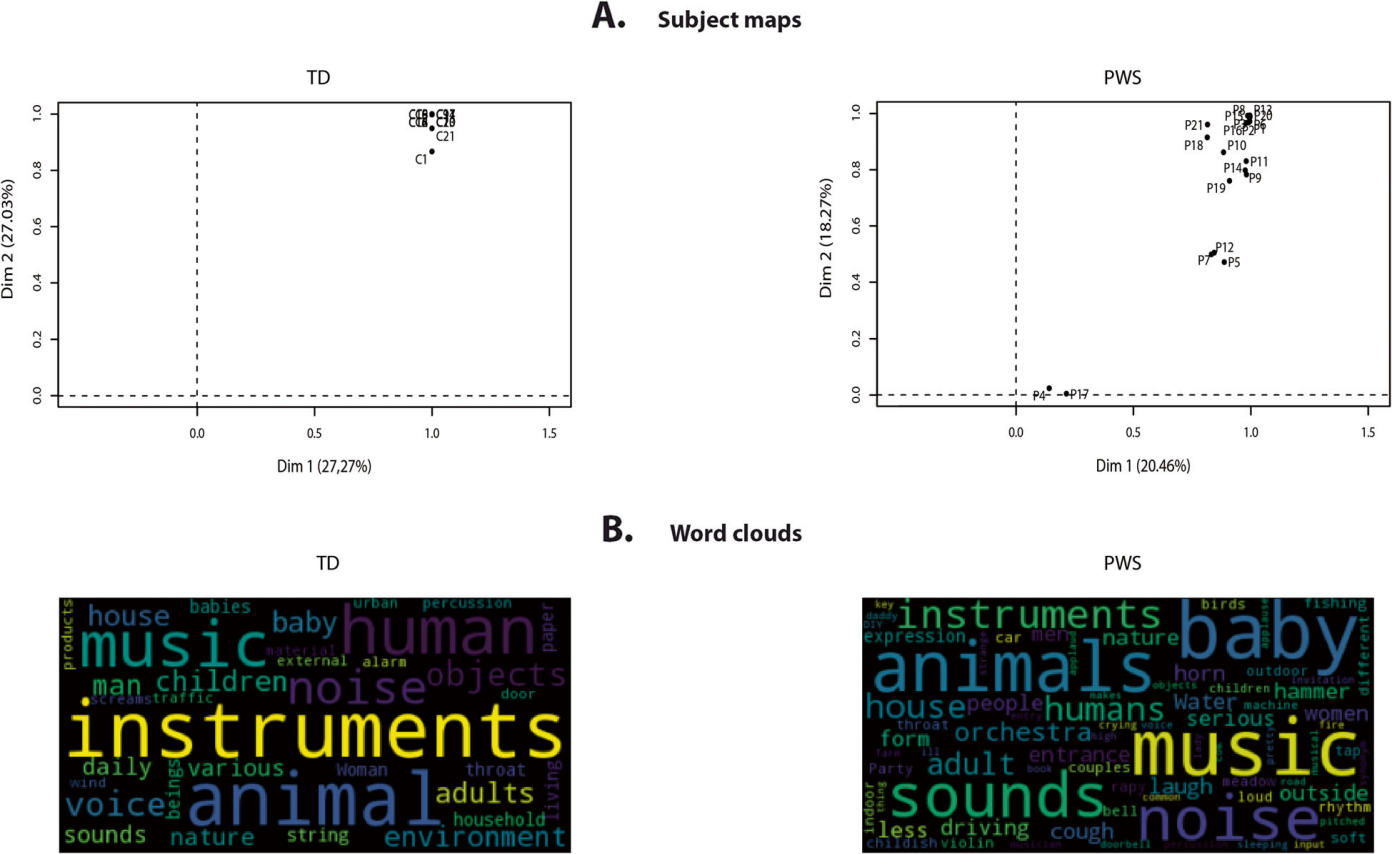
Participant maps and word clouds for sound categorization. Participant maps in **a** indicate the usage of the first two dimensions in the MCA maps by each participant and the homogeneity of categorization across PWS participants. In these maps, participants located above 0.8 made the greatest use of the given dimension. In **b**, the size of the words in word clouds reflect the frequency of their usage by the participants. These word clouds show that participants with PWS and controls produced broadly similar descriptions, the most frequently used words being ones relating to music and animals

This variability was confirmed when we analyzed the participants’ maps, which indicated the degree of homogeneity of categorization within each group (Fig. 4a). This representation demonstrated that all controls extensively used both the first and second classification dimensions, as all controls had values above 0.8 for each dimension. There was a rather different picture for PWS participants, as one subgroup (*n* = 2) did not use either of these two dimensions, while another subgroup (*n* = 3) extensively used the first dimension, but only moderately the second dimension. Of interest, the IQ scores of participants with PWS in these two subgroups were in the lowest range (45–51). However, when we looked at correlations between IQ, DBC, and dimension use, none of them was statistically significant. Nonetheless, Dimension 1 (separating musical from vocal sounds) was significantly correlated with PWS participants’ hit rate for voices (*r* = 0.55, *p* < 0.01), thus confirming that it corresponded to the separation of vocal from nonvocal stimuli, and that the PWS participants’ categorization strategy was based on their ability to discriminate between the two types of stimuli.

Lastly, we conducted a word cloud analysis of participants’ descriptions of their sound categories (Fig. 4b). These word clouds showed that participants with PWS and controls produced broadly similar descriptions, the most frequently used words being ones relating to music and animals. This clearly indicates that PWS participants categorized sounds on semantic not acoustic criteria. However, PWS participants also frequently used the words *sounds* and *noise*, which featured less prominently in the controls’ word clouds. This may reflect a lack of ability to produce precise verbal descriptions of the sounds.

## Discussion

### Social interactions and voice processing in PWS

Like individuals with ASD, participants with PWS display problems in social functioning, characterized by a reduced ability to interpret and respond to social information [4]. Their empathy deficit, combined with social withdrawal, prevents them from engaging in harmonious peer-group relationships [6, 18, 19]. It is only natural to assume that their social interaction difficulties are related to deficits in processing the two major sources of information in human communication: the human face and voice [20, 21]. Concerning facial information processing, participants with PWS have a known facial recognition deficit related to an altered strategy of face exploration [12]. Belin et al. [22] suggested that the human face and voice constitute a fused entity-in which case, face processing deficits should be accompanied by voice processing deficits. According to Salles et al. [11], participants with PWS present a specific deficit in distinguishing voices from nonvoices. However, it remains unclear whether their voice identification is also impaired and whether this is related to their performance for environmental sounds.

In the present study, we found that participants with PWS, especially UPD participants, were deficient in the recognition of voices and nonvoices. This deficit was slightly weaker for voices than for nonvoices in both PWS subgroups. It was accompanied by significantly longer RTs, with no difference between voices and nonvoices.

Given that no auditory deficit was reported for any of the PWS participants we tested, this voice recognition deficit is unlikely to be of sensory origin, except at an advanced level of sensory integration (e.g., multisensory integration). Salles et al. [11] demonstrated decreased multisensory benefits with an absence of violation of the race model indicating that multisensory information does not converge in participants with PWS. Though some of the participants were the same as in the present study, the analyses performed in the present study are different from those in Salle et al. [11], so that we cannot compare directly participants’ performances with Salles et al. [11].

Although this may depend on the particular task and the cognitive load, the participants with PWS seemed to have specific impairments that could not solely be explained by their ID. Even if top-down effects of impaired integrative functions cannot totally be excluded, the deficit in the perception of voice and nonvoice sounds was more likely to be attributable to impairment of the most integrative associative sensory areas (e.g. posterior portion of the superior temporal sulcus (STS) known to be involved in integrative and multisensory analysis, and temporal poles involved in voice processing). The temporal pole (Brodmann area, BA 38) was found to be hypoactive in a resting-state PET study of participants with PWS, as was the posterior temporal area (BA 22) [23]. Individuals with ASD also show deficient activation during voice perception in the temporal voice areas, which are typically more sensitive to vocal stimuli [8]. Moreover, there is a hypothesis that autism results from the disconnection of different brain areas owing to STS dysfunction [24]. A variety of sensory disabilities have been reported in ASD [25], and similar ones may be present in participants with PWS.

Hit rates showed that UPD participants were more impaired on voice and nonvoice perception than DEL participants. These findings confirmed that the participants with PWS had a sensory integration deficit, but also indicated that their higher order integrative deficits needed to be considered, given that PWS is characterized by ID and impaired social adaptation. To unravel these effects, we looked for correlations between hit rates for voices and nonvoices and IQ and DBC scores, but no significant correlation was found.

To further check whether this difference could be due to ID, we ran a Mann-Whitney test to analyze the difference in IQ between UPD and DEL participants, but found that it was nonsignificant (*p* > 0.6). It is therefore unlikely that the differences between the UPD and DEL participants on voice and nonvoice identification were related to ID. This confirms the specific deficit of participants with PWS for voice/nonvoice discrimination, but also their heterogeneity [11], and explains the identification results we found.

Although participants with PWS had a voice perception deficit that could be predicted from their impaired social functioning, their deficit for nonvoices was even more pronounced. This finding may contradict the hypothesis of a centrally driven, highly integrative origin of the deficit, insofar as voices require a more integrative cognitive function related to the perception of identity and personality [26]. Then again, PWS participants’ compensatory mechanisms for the recognition of such socially important stimuli as voices could be of central origin. However, given the social deficits of these PWS participants, it would be difficult to attribute this compensation for voices to social feedback or social adaptation, as opposed to the special role of the voice.

### Decision Modelling with HDDM

The longer RTs for voice and nonvoice detection in PWS (Fig. 1b) raise the question of whether they were due to slower decision-making or to a general slowdown in perception and motor reactions. To address this question, we used the HDDM, which implies that before giving a response, individuals have to accumulate and integrate a certain amount of information. The precise amount of information they need to arrive at a decision is represented by a threshold, while the speed at which they reach this threshold is the drift rate. Importantly for our question, the model also deduces their nondecision time, reflecting the time it takes them to execute the motor control and detect the stimulus. We assumed that PWS participants’ nondecision time and drift rate would both be longer, owing to their general slowdown.

For both voice and nonvoice identification, participants with PWS exhibited a similar pattern of parameters in the HDDM. They had a higher threshold for both types of auditory stimuli, meaning that they needed to accumulate more information to identify them than controls did. This need for more information can be explained by a lack of integrative brain capacity, linked to their general ID. This alone would have been enough to slow down their responses, but they were also slower at accumulating the necessary information (lower drift rate).

As a results, both factors (higher threshold and lower drift rate) contributed to the long RTs of participants with PWS, which were nearly twice as long as those of controls (Fig. 1b). Moreover, PWS participants had longer nondecision times than controls (Fig. 2b), which also contributed to their longer RTs.

Thus, the HDDM demonstrated that a number of different processes contribute to the behavioral slowdown in participants with PWS. Furthermore, the initial bias parameter indicated that participants with PWS were predisposed to the perception of voices, but to a lesser extent than controls were. It is curious that, despite the significant difference in performance between the UPD and DEL participants, the HDDM did not indicate any difference between the two subgroups on any of the parameters. This may mean that the UPD and DEL participants used similar cognitive strategies, but were more or less efficient in doing so, leading to the significantly different performances. According to the HDDM, participants with PWS needed more time to accumulate information for decision-making and were predisposed to voice perception. The sound categorization tests highlighted categorization strategies similar to those of controls, although PWS participants had more problems describing the categories they had created.

### Auditory free sorting task

Our exploration of PWS participants’ performances on the identification of predefined categories of voices and nonvoices led us to postulate that the deficit we observed was not related to higher-order cognitive functions, but instead to a deficit in integrative sensory processing in the temporal lobes. To further verify this hypothesis, we examined the results of an FST task that required greater involvement of cognitive and intellectual abilities such as similarity judgment, working memory, and executive functions [27]. The additional load on high-order integrative functions was generated by requiring participants to establish the categorization criteria/principles for themselves. In an FST, participants may group items according to a variety of subjective criteria, but sounds are usually grouped according to their common semantic or acoustic properties [16, 28, 29]. We found that controls divided the sounds they heard into voice, instruments and environmental categories (i.e., categorization predominantly based on semantic information as a consequence of identifying the sources of the sounds). Both the tree diagrams (Fig. 3A) and MCA maps (Fig. 3b) showed that participants with PWS created the same voice, instruments and environmental categories. This means that participants with PWS used the same semantically based cognitive strategy as controls. Furthermore, no correlations were found between categorization and IQ. PWS participants’ IQ therefore only weakly influenced their ability to establish sound categories. This weak influence could be detected at the within-category level, where the within-group distances between stimuli were significantly greater than they were for controls. The within-category dispersion can be explained by subgroups of PWS participants with low IQ who did not categorize the sounds as the other PWS participants did. The outlier PWS participants in the participant maps (Fig. 4a) had a lower IQ than the other PWS participants (there were no outliers in the control group). However, the correlation with IQ disappeared when we considered it from the opposite direction, in that not all PWS participants with low IQ were outliers with poor categorization performances.

As demonstrated by the word clouds (Fig. 4b), participants with PWS were less accurate in the description of the stimuli because of their poorer vocabulary, which may have been related to their ID. We also noticed that they tended to tell stories involving the stimuli, instead of providing an exact description of each category they formed.

As previously discussed, the HDDM indicated that participants with PWS needed more time to accumulate information to make a decision. Higher information accumulation demands may explain their relatively good results on categorization, where no time limits were imposed. This observation evokes the theory that ASD is the phenotypic expression of spatiotemporal processing disorders, which may result from multisystem brain disconnectivity-dissynchrony, defined as an increase or decrease in functional connectivity and neuronal synchronization within/between multiple neurofunctional territories and pathways [30]. Consequently, the world changes too fast for these participants, but given enough time, their brain can find compensatory pathways and circuits.

### Differences between UPD and DEL participants

Hit rates indicated that UPD participants had poorer voice and nonvoice perception than DEL participants (Fig. 1a). Similarly, UPD participants had longer RTs for both voices and nonvoices (Fig. 1b). This is in line with the finding of Salles et al. [11] that UPD participants have a greater deficit for the discrimination of voices and environmental sounds than DEL participants. However, the HDDM did not reveal any differences between the UPD and DEL participants on the decision making parameters. For sound categorization in the FST (Fig. 4a), three of the five PWS participants with the poorest performances were DEL participants, and the remaining two were UPD participants, so no conclusion can be reached as to possible differences between these subgroups. This may mean that UPD participants had more problems with the explicit task and fewer problems with the more implicit FST. Considering the absence of differences on the FST and the HDDM for decision-making, our overall results suggest that the differences in voice and nonvoice perception between the UPD and DEL participants concerned integrative sensory processing rather than the higher cognitive functions related to decision-making and ID.

## Conclusions

In this study, we found a deficit in participants with PWS for voice processing, but UPD participants were more impaired than DEL participants on both voice and nonvoice perception. We were also able to demonstrate a compensatory improvement in the perception of voices compared with nonvoices. The HDDM enabled us to demonstrate that participants with PWS need to accumulate more information for decision-making, are slower at decision-making, and are less predisposed to voice perception than TD individuals. Sound categorization in participants with PWS is generally preserved, though impoverished, and may be influenced by their low IQ.

## Methods

### Participants

Participants were 38 TD adults (mean age = 30 years, *SD* = 5) and 61 individuals with PWS (*M*_age_ = 30 years, *SD* = 7): 38 with DEL and 23 with UPD. PWS participants were initially assessed either at Hendaye Hospital (*n* = 26), a dedicated rehabilitation center for adults with PWS, or during a consultation at the PWS reference center of Toulouse University Hospital (*n* = 35). The present study is an extension of the Salles et al. [11] article, in the present set of analysis we have included some participants from the previous study. However, not all of the previous participants performed the totality of the tests analyzed in the present study and the majority of PWS participants in this study did not overlap with the study of Salles et al. [11]. See Table 1 for the participants’ data.

**Table 1:** Summary description of study participants

	Age	M	F	DEL	UPD	IQ	DBC
Mean PWS (*SD*)	30 (7)	29	32	38	23	56.6	0.32
Mean TD (*SD*)	30 (5)	16	22				

*Note. DEL* deletion on chromosome 15, *UPD* uniparental disomy, *IQ* intelligence quotient, *DBC* Developmental Behavior Checklist, *PWS* participants with Prader-Willi syndrome, *TD* typically developing controls

The study was approved by the ethics committees of Toulouse University Hospital (Toulouse Hospital CHU 13687203; National EudraCT 201,300,437–33), and all participants gave their written informed consent prior to their inclusion in the study.

### Clinical assessment

The Developmental Behaviour Checklist for Adults (DBC_A) is a questionnaire completed by parents or caregivers to assess the behavioral and emotional problems of adults with developmental and intellectual disabilities, and it is routinely used for participants with PWS. The full questionnaire contains 107 items divided into six categories: disruptive/antisocial, communication disturbance, anxiety, self-absorbed, depressive, and social relating.

### Voice discrimination task

We assessed participants’ ability to distinguish between vocal and nonvocal stimuli in a two-alternative forced-choice (2FAC) paradigm. Each participant sat in a quiet, dimly lit room looking at a fixation cross on a computer screen. They were tested with a 1-s intertrial interval and were instructed to respond as accurately as possible, using the left or right control button of the E-prime response box to indicate their answer (voice or nonvoice). The response keys were counterbalanced across participants, and they each underwent a short training session to ensure that they understood the test. The 110 stimuli were presented in two blocks of 55.

All the stimuli were taken from a database containing vocal and nonvocal sounds used in previous experiments [11, 31, 32]. They each lasted 500 ms. The set of 55 vocal stimuli included 29 speech stimuli (phonemes presented in a /h/−vowel−/d/ context, words in different languages, or nonsemantic syllables) and 26 non-speech stimuli (e.g., laughs, coughs). The set of 55 nonvocal stimuli consisted of a wide variety of environmental sounds (cars, telephones, bells, running water, etc.). Neither set contained animal vocalizations.

### Auditory free sorting task

Most studies exploring how we categorize natural sounds are based on pairwise similarity judgments, but one alternative method of determining how natural sounds are perceived is to use an FST. This task provides an opportunity to test a large set of stimuli without having to divide them into dimensions beforehand, thus allowing participants to categorize them according to their own criteria/principles. The FST has been shown to be well-suited to evaluating auditory perception in adult participants, as well as in children as young as 6 years [16, 28, 29]. In an FST, participants group the objects according to their common semantic or acoustic properties. Although this free categorization process is closely related to similarity judgment, the process involves more holistic-based decisions [33] and is more strongly influenced by cognitive factors [27]. In the present FST categorization protocol, both groups were seated in front of a PC monitor positioned at eye level, with loudspeakers located on either side at a distance of 1 m. The stimuli were played at a level of 65 dB SPL (measured at head height with a sound level meter at a distance of 1 m) through loudspeakers in free-field listening conditions. Testing was carried out using open-source TCL-LabX software (http://petra.univ-tlse2.fr/tcl-labx/), which acted as the interface for the FST. The 16 sounds were represented on the computer by 16 numbered and colored squares that were positioned in the same order for all participants.

The task for participants was to listen to the 16 sounds and place them in groups (i.e. create categories) using any criteria they chose. The experimenter gave only minimal feedback to facilitate completion of the experiment. Sounds were played using the PC mouse, by double clicking on each square, and participants created categories by dragging and grouping the squares together on the screen. Once participants had finished placing the squares in categories, they were asked to listen to each sound one last time to verify their choices before ending the experiment. They were then asked to type a brief description of each category using the keyboard.

There were no limits on the amount of time taken to complete the test or the number of times participants could listen to a given sound (i.e., playbacks). Participants were also allowed to create as many or as few categories as they wished, such that one category could contain just a single stimulus or all 16. The TCL-LabX software also recorded performance data and statistics for all participants, including the number of categories they created, the number of playbacks they listened to, and the duration of the experiment.

All the sounds were taken from a database owned by the PETRA group at Toulouse Jean Jaurès University (http://petra.univ-tlse2.fr) and were chosen to cover a broad range of semantic and acoustic information (see [28]). We selected sounds that are frequent in everyday life and can be divided into three main types: environmental sounds (alarm clock ringing, car engine starting, door opening, footsteps, glass breaking, helicopter, running water); musical sounds (bells, guitar, oboe, violin, xylophone); or vocal sounds (male voice coughing, female voice speaking, female voice laughing, male voice speaking). Stimuli were presented at the comfortable level of approximately 65 dB SPL and were delivered in stereo through headphones plugged into the computer.

### Data analysis

The participants’ performances on the voice discrimination task were analyzed in the form of hit rates and RTs (Fig. 1), using the general linear mixed-effect model of the lme4 R package, with the factors group (TD, Del PWS, UPD PWS) and stimulus (vocal, nonvocal) and the Group x Stimulus interaction. We ran type II Wald chi-square tests for post hoc comparisons.

Hit rates and RTs were then analyzed with the HDDM [13], a sequential sampling model that correlates response accuracy with RTs for simple 2FACs. It postulates that each decision can be modulated by the accumulation of noisy information over time. Occurrences accumulate until they reach a threshold when the individual takes a decision. Each decision is represented by an upper and a lower boundary that have to be crossed in order to initiate the corresponding response. Applying the Bayesian approach to the HDDM can shed light on the cognitive and psychological processes behind decision-making, based solely on RT distribution for the two response choices. With this model, the behavioral data can be categorized according to four parameters (see Fig. 2a): *threshold*, *drift rate* for the accumulation speed, *nondecision time* associated with stimulus perception and response execution, and *initial bias*. We used the Monte Carlo and Markov chains (MCMC) method to estimate posteriors based on our data. We performed 20,000 iterations. We discarded 5000 initial burn-in items, and only saved every fifth sample. This method yielded 3000 posterior values that were normally distributed. We confirmed our model using the posterior plots available in Python software (PyMC). From this simulated population, we could calculate the mean and 95% confidence interval for each parameter.

In the categorization part of the study, to analyze the sound categories the participants created, we applied two approaches in the R environment [34]: hierarchical clustering based on principal components (HCPC) allowed us to represent stimulus associations as tree diagrams; and multiple correspondence analysis (MCA) allowed us to obtain the group-level statistics for the preferred associations of stimuli.

More specifically, we performed HCPC in order to view a simplified version of the sound categories in the form of tree diagrams. With this analysis, it is not possible to account for all of the variance (inertia) within the data (i.e. the variability of participant responses), and so a certain proportion remains unaccounted for. However, by increasing the number of desired categories, the inertia can be reduced, and it was by using this process that we were able to choose the final number of categories: if the number of categories is Q, then the optimum number of categories is found when the change in inertia is greater when moving from Q - 1 to Q than from Q to Q + 1 [34].

We applied MCA to a multi-participant categorization table (raw data not included) produced by TCL LabX software. This table represented the results as an array of categorical variables as columns and categorical items (sound stimuli) as rows, with each cell containing a number that defined the category membership of each sound for each participant. MCA used correspondence analysis to represent each sound as a data point in an *n*-dimensional Euclidean space based on the categorical values (i.e., categories created by participants). Each of the dimensions was chosen to account for the greatest amount of variance possible within the dataset, and they were produced in descending order of variance. MCA on the participants showed how strongly individual results coincided with the dimensions [35]. A total of 15 dimensions were used in the analysis. We focused on the two most significant ones (Dim 1 & Dim 2), as they accounted for the greatest amount of variance in the data and also showed the most significant correlations with the acoustic variables measured for the sounds. As there was no a priori knowledge that could be used to automatically establish these relations, a degree of interpretation was required when commenting on the dimensions [35].

To characterize the distances between the sounds in the MCA maps, we calculated the corresponding Euclidean distances.

## Data Availability

The datasets used and/or analysed during the current study are available from the corresponding author on reasonable request.

## Abbreviations

2FAC: Two-alternative forced-choice task
ASD: Autism spectrum disorder
DEL: Deletion
FST: Free sorting task
HCPC: Hierarchical clustering based on principal components
HDDM: Hierarchical drift diffusion model
MCA: Multiple correspondence analysis
MCMC: Monte Carlo and Markov chains
PWS: Prader-Willi syndrome
RT: Reaction times
TD: Typically developed
UPD: Maternal uniparental disomy
